# Genetic diversity and divergence applied to Enviromental services for *Araucaria angustifolia* (Brazil)

**DOI:** 10.1186/1753-6561-5-S7-P9

**Published:** 2011-09-13

**Authors:** Luciano Medina-Macedo, Juliana Bittencourt, Carlos Soccol, Andre Lacerda

**Affiliations:** 1 Biotechnological Processes, Federal University of Parana, Curitiba, Parana, 81531-990, Brazil; 2 Federal Technological University of Parana, Ponta Grossa, Parana, 84016-210, Brazil; 3 EMBRAPA FLORESTAS, Colombo, Parana, 83411-000, Brazil

## 

Humans derive many utilitarian benefits from the environmental services of biotas and ecosystems.Yet ecosystem, species and genetic levels is increasingly lost from agricultural landscapes mainly due to maximisation of production [1,2]. The genetic variability is a result of several forces including mutation, recombination and gene flow. Allele frequencies are altered by natural selection and by genetic drift. Whilst mutation, recombination and genetic drift are random and independent processes, natural selection is a directional process towards evolutionary change [3]. The Araucaria Rainforest is one of the most important biomes occurring naturally in southern Brazil. The extensive logging and agricultural expansion became this forest extremely fragmented. Particularly important is the factor of ecosystem resilience, which appears to underpin many of the services. While biodiversity often plays a key role, the services can also derive from biomass and other attributes of biotas, for example, genetic diversity of a particular species. Landscape genetics are being used in this project to improve the understanding of fragmentation impact to assist an environmental services scheme. The objective of this study was to assess by microsatellites markers, the genetic diversity and dynamics in remnant patches of *Araucaria angustifolia* rainforest, with different levels of human modification. The project compared two different forest conditions: one is 1.157,48 ha of continuous forest, and the other is a fragmented forest remnant, with size from 8 ha, appraised 5 km from the continuous forest area. Genetic diversity and divergence of seedlings and adult individuals present in forest fragment was compared with the genetic composition of samples appraised in continuous forest. Cambium and seed material were collected from each recorded tree, and genomic DNA was extracted using the method described in Mazza & Bittencourt (2000). The seeds for DNA extraction had undergone a slight adjustment – Proteinase K was added to megagametophyte (maternal origin) and embryo extraction. Polymerase chain reaction (PCR) conditions used were establish by Qiagen Multiplex Master Mix protocol (1022830) and use fluorescents dye labelling. Two multiplexed systems of microsatellites were applied with the 8 primers. The eight loci used were CRCAc2, Ag23, Ag62, Ag45, CRCAc1, Ag20, Ag56 and As90. Following PCR this dilution was running in ABI sequencer 3100. Gene Scan and Genotyper software were used for data collection and alleles analysis. The average number of alleles per locus among adult trees in the continuous forest was 8.10, compared to 5.15 appraised in forest fragment. The means of expected heterozygosity were 0.623 for continuous forest and 0.579 for forest fragment, while observed heterozygosity range was respectively, 0.571 and 0.723. Subpopulations of forest fragments were more distinguished than subpopulations of continuous, due the average fixation index was 0.082 for subpopulations in the continuous forest and 0.210 in fragment. It is worth noting that the levels of genetic differentiation among all subpopulations can be considered to be high. Paternity analysis, within the continuous forest indicated that 48% of offspring were fertilized by pollen from trees outside the plot site. The average pollination distance within the continuous was 95 m. In the trees from forest fragment, the analysis showed that 42% to 65% of the offspring was fertilized by pollen from trees outside fragment. The effective number of pollen donors in the continuous forest ranged among seed-trees from 2 to 10, and in the fragment from 2 to 6. The results suggest high pollen dispersal distance in both conditions and an absence of reproductive isolation. They also show high pollen immigration and dispersal distance among the tree groups. The results suggest that fragmentation increases divergence in *Araucaria angustifolia.*The population in continuous forest showed higher genetic diversity in the adult population than the population of trees in fragment. The reducing the heterozygosity were low, may be due the recent forest fragmentation history. There are more inbreeding in fragmented population than in continuous population. Fragmentation increased the genetic divergence among the fragmented population. However, the genetic results indicated the presence of long-distance dispersal leading to functional connectivity between isolated forest fragment. The survival of remnants of *Araucaria angustifolia* patches as well as single trees in the agricultural landscape is key factors for species conservation and could be applied to environmental payment services, these strategy for *A. angustifolia* is an integration of conservation strategies across reserves and the surrounding matrix, including productive agricultural areas, to assist gene flow movement between temporally suitable habitats.
